# A shortcut to sample coverage standardization in metabarcoding data provides new insights into land-use effects on insect diversity

**DOI:** 10.1098/rspb.2024.2927

**Published:** 2025-05-07

**Authors:** Mareike Kortmann, Anne Chao, Chun-Huo Chiu, Christoph Heibl, Oliver Mitesser, Jérôme Morinière, Vedran Bozicevic, Torsten Hothorn, Julia Rothacher, Jana Englmeier, Jörg Ewald, Ute Fricke, Cristina Ganuza, Maria Haensel, Christoph Moning, Sarah Redlich, Sandra Rojas-Botero, Cynthia Tobisch, Johannes Uhler, Jie Zhang, Ingolf Steffan-Dewenter, Jörg Müller

**Affiliations:** ^1^ Chair of Conservation Biology and Forest Ecology, Julius-Maximilians-Universität Würzburg, Würzburg, Germany; ^2^ National Tsing Hua University, Hsinchu, Taiwan; ^3^ Department of Agronomy, National Taiwan University, Taipei, Taiwan; ^4^ Department of Conservation and Research, Bavarian Forest National Park, Grafenau, Germany; ^5^ Advanced Identification Methods GmbH (AIM), Lepizig, Germany; ^6^ Division of Biostatistics, University of Zürich, Zürich, Switzerland; ^7^ University of Applied Sciences Weihenstephan-Triesdorf, Freising, Germany; ^8^ Department of Animal Ecology and Tropical Biology, Julius-Maximilians-Universität Würzburg, Würzburg, Bayern, Germany; ^9^ Professorship of Ecological Services, University of Bayreuth Bayreuth Center of Ecology and Environmental Research, Bayreuth, Bayern, Germany; ^10^ University of Applied Sciences Weihenstephan-Triesdorf Institute of Horticulture, Freising, Bayern, Germany; ^11^ Restoration Ecology, TUM School of Life Sciences, Freising, Bayern, Germany; ^12^ Institute of Ecology and Landscape, Weihenstephan-Triesdorf University of Applied Sciences, Freising, Bayern, Germany

**Keywords:** Hill numbers, taxonomic diversity, phylogenetic diversity, phylogenetic tree, biodiversity, climate change

## Abstract

Identifying key drivers of insect diversity decline in the Anthropocene remains a major challenge in biodiversity research. Metabarcoding has rapidly gained popularity for species identification, yet the lack of abundance data complicates accurate diversity metrics like sample coverage-standardized species richness. Additionally, the vast number of taxa lacks a unified phylogeny or trait database. We introduce a new workflow for metabarcoding insect data that constructs a phylogenetic tree for most insect families, standardizes sample coverage and assesses both taxonomic and phylogenetic diversity along the Hill series. Applying this workflow to Central Europe, we analysed insect diversity from 400 families across a land-use gradient. Our results show that land-use intensity significantly affects sample coverage, highlighting the necessity of biodiversity standardization. Taxonomic diversity declined by 27–44% and phylogenetic diversity by 13–29% across 39 000 operational taxonomic units, with diversity decreasing from forests to agricultural areas. When focusing on rare species communities exhibited greater phylogenetic diversity loss than taxonomic diversity, whereas dominant species experienced smaller phylogenetic losses but more pronounced declines in taxonomic diversity. Our findings underscore the detrimental effects of agriculture on insect taxa and reveal a dramatic loss of phylogenetic diversity among rare species with potential consequences for ecosystem stability.

## 1. Introduction

Evidence of declining insect populations has alarmed both the scientific community and society [1,2]. However, the complex interactions among various factors potentially responsible for the decline in different biodiversity metrics of insect communities continue to fuel controversial debates [3–6].

During the last decade, many studies have analysed the temporal trends of insect declines in different regions of the world. One of the most prominent studies by Hallmann *et al*. [7] showed a drastic decline in insect biomass in protected areas in Central Europe. Neff *et al*. [8] also observed that climate and land-use changes affect the decline of insect species distributions in butterflies, grasshoppers and dragonflies. Similarly, a temporal analysis of bumblebees in Europe showed that suitable habitat will likely be reduced by at least 30% due to climate change and land use [9]. However, trends in insect populations are highly heterogeneous among different taxa and environments and depend on the target metric like biomass, abundance or diversity measures [10–12]. Recent studies showed the importance of considering the different characteristics of insect communities to gain a better understanding of the ongoing changes in insect populations. For example, van Klink *et al*. [12] showed that the most severe temporal decline in the abundance of terrestrial insect communities occurs in dominant and not rare species.

## 2. Introducing a workflow for comprehensive diversity measures

One significant challenge in studying insect decline stems from their vast diversity. To efficiently identify such a broad range of taxa, metabarcoding has become increasingly prevalent. However, sequencing data lack reliable abundance data equivalent to individual counts, because the number of individuals in metabarcoding samples cannot be reliably inferred from sequencing reads. Therefore, most studies rely solely on observed species numbers (often erroneously referred to as species richness instead of species density [13]), ignoring frequency distributions. This approach has the following two distinct limitations: the inability to standardize for varying levels of sample coverage and the inability to analyse more meaningful diversity metrics, such as species richness [13], beyond mere species counts. Moreover, the sequence data obtained through metabarcoding often remain underutilized, despite their potential to provide valuable insights into phylogenetic relationships [14,15].

Sampling of insects or other diverse groups usually leaves some species undetected. Hence, insect samples are generally incomplete [16]. However, the degree of completeness can vary greatly and systematically between habitat types, sampling methods and species communities. Therefore, scientists have developed frameworks to calculate and control for sample coverage (or simply coverage), an objective measure of sample completeness. Coverage is defined as the proportion of individuals or frequencies in the entire community belonging to species detected in a sample.

We have set-up a workflow that allows us to consider taxonomic and phylogenetic diversity, but also the variation in sample coverage across habitats, an aspect that has been neglected by most previous analyses of insect diversity trends (but see van Klink [12]). Contrary to most people’s intuition, Alan Turing’s cryptographic work showed that the sample coverage of a sample can be very accurately estimated based on the number of singletons. His work can easily be transferred to an ecological context, in which a singleton refers to a species or other taxonomic entity that is only observed with one individual or another single frequency count within a sample [17]. Still, to calculate sample coverage, it is necessary to be able to effectively use frequency distributions of metabarcoding, which is still an open area of research [18–20]. We propose that we can use the number of reads generated during sequencing to describe the frequency distribution of species or other taxonomic units within a sample, after estimating the true number of singletons in the sample based on Turing’s wisdom [17,21] on statistical methods [18]. Including these methods our workflow will allow to standardize for differences in sample coverage, an important step to control for biases by different habitat types, sampling years or others.

Nevertheless, even after controlling for sample coverage, we still have different species frequency distributions within our observed communities or samples. To incorporate species frequency distributions into biodiversity measures, a consensus has emerged among ecologists in biodiversity research that Hill numbers [22] (effective number of species) should be used to quantify species or taxonomic diversity (Ellison [23] and subsequent papers). Hill numbers allow us to calculate diversity values with focus on rare species (*q* = 0 or species richness), common species (*q* = 1 or Shannon diversity) and dominant species (*q* = 2 or Simpson diversity).

It is important to note that the Hill numbers in this study are based on metabarcoding reads, which reflect a combination of the number of individuals sampled, their biomass and taxon-specific amplification biases, even after controlling for differences in insect size through sieving (see §4). Still, we hypothesize that metabarcoding reads can be used as an approximation for abundances when analysing entire samples and not making taxon-specific comparisons. Chiu & Chao [18] established the validity of using such measures for microbes, which face similar challenges in quantifying abundance. Their method improves the reliability of diversity estimates and ensures fair comparisons across communities by estimating the true number of singletons and standardizing each sample. To avoid ambiguity, the term ‘frequency distributions’ will be used to refer to abundance distributions.

In addition to taxonomic diversity, functional diversity is a widely accepted metric to better understand ecosystem functioning and resilience [24]. However, due to the absence of a consistent method or database for analysing functional traits across all terrestrial insect families [25], we used phylogenies as a practical proxy [26]. Because species traits are shaped by evolutionary processes, closely related species often share similar functions and traits [27], and while deviations from this pattern exist, functional and phylogenetic diversity are highly correlated [27]. To date, no comprehensive phylogeny includes the majority of insect families while resolving to the species or operational taxonomic unit (OTU) level, which limits the analysis of metabarcoding data (but see Chester [28]). To overcome this limitation, we incorporated a phylogenetic tree calculation into our workflow, allowing for the analysis of large numbers of OTUs in metabarcoding samples. This workflow integrates a backbone tree of insect families with genetic sequences from metabarcoding. The backbone tree, which captures deep evolutionary lineages [29], provides a structural framework, while CO1 sequences from metabarcoding offer finer taxonomic resolution within insect families.

The entire workflow including the singleton filter, the insect phylogeny and subsequent standardized diversity calculations introduces a novel approach for calculating phylogenetic and taxonomic diversity measures in hyperdiverse insect samples. Importantly, it standardizes comparisons across samples with varying coverage, facilitating the analysis of species richness (*q*0), Shannon diversity (*q*1) and Simpson diversity (*q*2). Within this unifying framework of Hill numbers, we can also directly compare diversity measures for all numbers of *q* and between taxonomic and phylogenetic diversity [30].

## 3. Analysing impacts of land use, climate and weather on insect communities

We applied this workflow to quantify the effect of land use (hypothesized as a major driver of insect decline [2]) on overall insect diversity in Central Europe. For the first time, our approach accounts for the mathematical requirements necessary for accurately estimating diversity from large bulk samples using metabarcoding [13,17]. Specifically, we disentangle the effect of land-use intensity at both regional and local scales on taxonomic and phylogenetic insect diversity, while controlling for climate (long-term weather over 30 years) and short-term weather variability (day-to-day atmospheric conditions). To achieve this, we used the LandKlif study design, situated within a cultural landscape in Bavaria, Germany. This study encompasses five climatic zones, four distinct local land-use types (forest, grassland, arable land and settlement) and three regional land-use types (semi-natural, agricultural and urban), each representing increasing levels of land-use intensity [31].

## 4. Material and methods

### (a) Study area

We based this study on data from the LandKlif project (https://www.landklif.biozentrum.uni-wuerzburg.de), in Bavaria, Germany. A detailed description of the study design can be found in Redlich *et al*. [31]. Based on a grid cell system covering all of Bavaria, we selected 60 grid cells (quadrants), creating independent gradients of land use and climate. We based regional land-use types at the quadrant level, classifying them into three categories based on Corine land cover data from 2012: semi-natural regions were dominated by natural vegetation and forest; agricultural regions had at least 40% arable land; and urban regions comprised >14% urban areas. Therefore, each regional land-use type contained a higher proportion of each respective land use than the region-wide average. Given that quadrants were classified in three land-use types and five climate zones (using the multi-annual mean temperature of the period 1981−2010, according to the German meteorological service, Deutscher Wetterdienst, as cited by Redlich *et al*. [31]), 60 quadrants were selected to have 15 climate-land-use combinations, each with four replicates. Within each quadrant, three survey plots were selected based on the three most common land-use types of the quadrant. The local land-use types considered in this study were forest, grassland, arable land and settlement. This resulted in 179 plots with a spatial extent of 480 km^2^. Detailed maps of the study design can be found in Redlich *et al*. [31] and Uhler *et al*. [32].

### (b) Insect sampling

We installed one Malaise trap at each plot centre in 2019. Malaise traps were based on the Townes Malaise trap model with a black roof and slightly smaller size [33]. We used ethanol (80%) as the trapping fluid to ensure the preservation of DNA for barcoding. Traps were activated from mid-April to mid-August and emptied every two weeks, resulting in eight sampling campaigns and a total of 1259 insect samples. The samples contained 41 489 OTUs of insects, of which 39 113 OTUs were assigned to 400 different insect families. Detailed information on insect sampling can be found in Uhler *et al*. [32].

**Figure 1.**
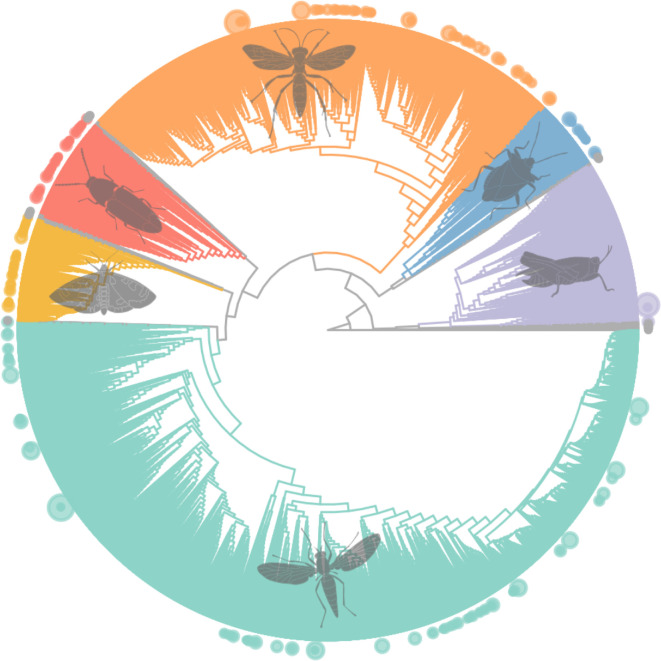
Phylogenetic tree of 39k OTUs from 400 insect families and their diversities. The main insect orders are depicted in colour (Hymenoptera in orange, Hemiptera in blue, Orthoptera in lilac, Diptera in aquamarine, Lepidoptera in yellow and Coleoptera in red). The remaining smaller orders are depicted in grey. The dots at the end of the branches represent families, with the dot size indicating the number of OTUs within each family. The tree was calibrated with fossil calibration points from Rainford *et al*. [29].

Malaise traps capture the broadest range of insect taxa in terms of species and families. Although they focus primarily on flying insects, they provide a valuable link between pitfall traps and flight interception traps by covering both ground-dwelling and flying insects. Since Malaise traps collect a wide spectrum of Diptera and Hymenoptera (figure 1), which are difficult to identify visually, they are often paired with metabarcoding for species identification.

We separated the samples into smaller and larger insects using an 8 mm sieve to improve barcoding results [19]. When processing bulk samples, larger, biomass-rich specimens contribute disproportionately more DNA than smaller organisms, leading to imbalanced sequencing results. Previous research [34] showed a strong linear correlation between specimen biomass and sequencing read abundance, indicating that taxa with higher biomass dominate the read pool. Consequently, without size sorting, a few large specimens can overwhelm the dataset, necessitating deeper sequencing to detect smaller and rarer taxa. Hence, size sorting or sieving can improve the detection rate [19]. Separation into smaller and larger individuals also allows to control for differences in biomass, improving the role of reads as a surrogate for species frequency distribution within samples.

Species were identified using CO1-5P (mitochondrial cytochrome oxidase 1) DNA metabarcoding following the laboratory and bioinformatics pipelines reported by Hausmann *et al*. [35], which results in OTUs (for most orders, the mean OTU number per plausible species ranges between 1 and 2 [36]) and associated read counts. Quality filtering was conducted using the VSEARCH --fastq_filter utility, applying a maximum threshold of one expected error per sequence and retaining only reads with a minimum length of 300 bases (--fastq_maxee 1 --minlen 300). Following this, sequences within each sample file were dereplicated using the --derep_fulllength function, preserving only one instance of each unique sequence while appending size information and assigning new labels (--sizeout --relabel Uniq). Afterwards, the cleaned and de-replicated sample files were merged into a single FASTA file. This combined dataset was further dereplicated using --derep_fulllength and filtered to remove sequences that appeared only once across the entire dataset (i.e. dataset-wide singletons), using the parameters --minuniquesize 2 --sizein --sizeout --fasta_width 0. Further details on the metabarcoding pipeline are given in electronic supplementary material, S1 (metabarcoding).

### (c) Climate, local temperature and humidity

We recorded local temperature and humidity (collectively called ‘weather’) with ibutton thermologgers (type DS1923). At each site, we mounted one datalogger on a wooden pole, protected from direct sun exposure. We averaged hourly measurements of air temperature and relative humidity across the sampling periods. We calculated annual mean temperature and precipitation for each plot (from 1981 to 2010) based on gridded monthly datasets provided by Deutscher Wetterdienst with a horizontal resolution of 1 km (collectively called ‘climate’) [32].

### (d) Statistical analysis

**Figure 2.**
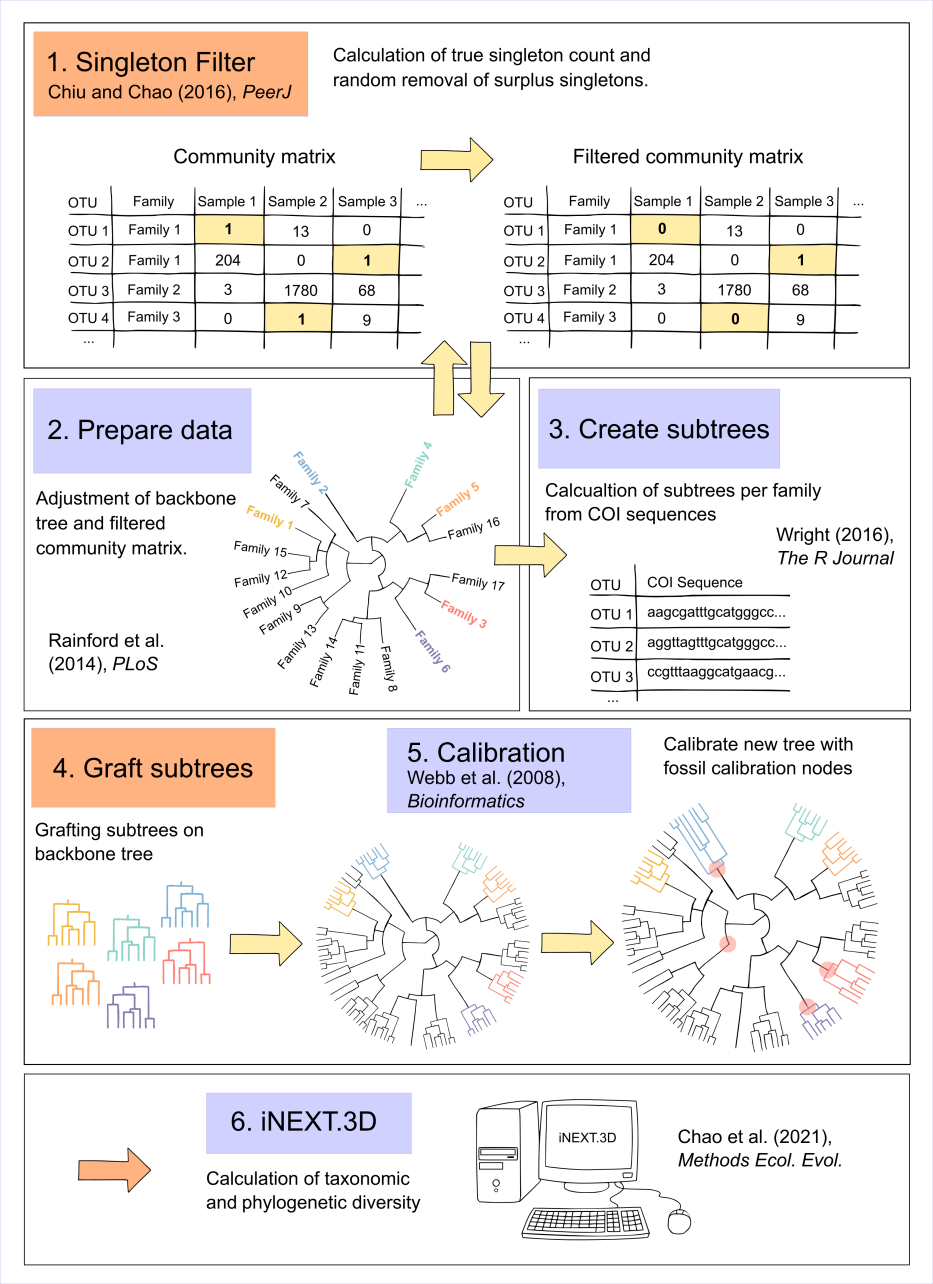
Overview of the new workflow. Our workflow includes a collection of previously published methods (blue), linked by new and updated code (orange). Newly developed code has also been used to combine the different methods. The workflow now allows us, for the first time, to analyse metabarcoding data with a phylogenetic tree in iNEXT (orange arrow). The numbers in the blue and orange boxes refer to the R scripts in the Zenodo repository [38].

We used 1259 insect samples for analyses of biodiversity and insect communities. All analyses were conducted in R 4.3.1 [37]. An overview of the newly developed workflow is presented in figure 2, with reference to the R scripts in theZenodo repository [38]. The workflow assembles existing methods, some of them with slight adaptations, and newly developed code to link everything together. The subsequent sections provide a detailed explanation of these methodologies.

### (e) Singleton filter

We first created a sample-OTU-read-matrix and used a procedure based on a method from Chiu & Chao [18] to control for sequencing errors, which typically lead to an inconsistent distribution of OTU observation frequencies (figure 2, step 1).

In their study, Chiu & Chao [18] addressed the challenge of inflated singleton counts in microbial diversity assessments. They developed a non-parametric estimator for the true number of singletons by analysing the observed frequencies of doubletons, tripletons and quadrupletons, assuming these counts to be reliable. This method is based on a modified version of the Good–Turing frequency formula, which relates the expected frequency of singletons to the frequencies of multiple observations. By applying this approach, the number of singletons can be corrected, leading to more accurate diversity estimates. A table with the calculated numbers of sequencing errors is given in the electronic supplementary material, S2. This correction was integrated into the Hill numbers framework to quantify the standardized alpha diversity of communities (figure 2).

We applied the singleton filter by randomly removing the calculated number of surplus singletons from each sample. Additionally, we slightly adapted the approach as in rare cases with sparse data, the original implementation could yield negative results and had to be adjusted. Since all filtering and standardization processes were performed within each sample, we used raw read counts rather than relative read counts. In summary, this improved method allows us to use read frequencies to describe species distributions within samples and to calculate sample coverage for metabarcoding data [18]. The enhanced version is available in R script 1.

To control for potential variation introduced by random filtering, we repeated the selection process and our analyses of phylogenetic diversity and community matrices five times. Since the different filtered matrices showed only minor variation in subsequent modelling, we retained a single matrix for our final results.

### (f) Phylogenetic tree

We built a phylogenetic tree based on metabarcoding sequences and a backbone tree by Rainford *et al*. [29]. Metabarcoding sequences are well suited to differentiate species, but they are not able to explain older phylogenetic divisions like the separation of different orders. To gain reliable information on older lineages, we used a dated phylogenetic tree of insects that covers relations down to families as backbone tree [29]. Although the backbone tree by Rainford *et al*. [29] is the most comprehensive for insect families to date, it can easily be replaced by newer or improved versions in our workflow, or even to other taxa identified by metabarcoding.

We added missing families to the backbone tree, but only when we found a scientific reference that provided a sister family that was already present in the phylogeny by Rainford *et al*. [29]. To resolve relations within families, we used the cytochrome oxidase subunit 1 (COI) sequences used to identify the insects in our data by metabarcoding. While COI has limitations for deep phylogenetic reconstructions due to high substitution rates and lineage sorting issues, it remains a widely used marker for taxonomic differentiations, species-level analyses and biodiversity assessments [39,40]. In our study, COI is primarily used to assess phylogenetic diversity within families, as its rapid mutation rate provides strong resolution at shallow evolutionary levels. Still, if better options arise in the future or other markers are used in other taxa, our code is flexible enough to incorporate other sequence types.

Sequences were aligned within each family using the *AlignSeqs* function from the *DECIPHER* package [41]. We calculated distance matrices for each alignment with the *DistanceMatrix* function from *DECIPHER* and estimated phylogenetic trees with the *TreeLine* function using the neighbour-joining method. Due to the extent of our data, we used neighbour-joining to prevent excessive calculation times. Our code allows also more complex models offered by *TreeLine*, like maximum likelihood. We advise to select the appropriate model for the data. Especially with smaller datasets, maximum likelihood can easily be applied and will result in more elaborated trees. The estimation of subtrees can be found in R script 3. The family subtrees were then added to the backbone tree at the respective family node with the *bind.tree* function from the *ape* package [42]. Due to the ultrametric structure of the backbone tree, family ages were then all the same, leading to skewed branches. To calibrate all branch length and allow consistent branch ordering, we used *make_bladj_tree* from datelife package [43], which uses the *bladj* function from *phylocom* [44]. As calibration points, we used the fossil data from Rainford *et al*. [29]. To ensure that the calibrated tree was ultrametric and suitable for utilization in iNEXT3D, the *forceEqualTipHeights* function from the *ips* package was employed. The assembly of the final tree can be found in R script 4 and the calibration in script 5.

### (g) Multiple regression of distance matrices

We calculated community distances for sample coverage standardized communities (SC = 0.996) along the Hill numbers for rare, common and dominant species based on Jaccard, Horn and Morisita–Horn indices using an adapted function based on the inext.beta3D function (R script 9 and 10), an approach by Chao *et al*. [45]. We calculated Euclidean distances for the mean day of sampling, climate and weather variables and geographic location (coordinates) using the *dist* function in R. For the categorical variables (i.e. local and regional land-use types), we calculated Gower distances using the *daisy* function from the *cluster* package [46,47]. Calculations of the distance matrices of environmental parameters can be found in R script 11. We calculated multiple regressions on distance matrices with the *MRM* function from the *ecodist* package [48] to test for effects of land use, climate and weather, day of the year and geographic location on differences in insect communities (R script 12).

### (h) Calculation of taxonomic and phylogenetic diversity

To estimate biodiversity measures, we used the *iNEXT.3D* package in R [49]. We used the *iNEXT3D* function to calculate the observed taxonomic and phylogenetic diversity as well as the sample coverage of each insect sample (R script 6).

Coverage-based standardization is a mathematically elegant and statistically robust way to standardize samples and has been increasingly used in the ecological literature [50]. Based on our data, sample coverage in 179 plots ranged from 0.981 to 0.999 (electronic supplementary material, figure S1.1). For highly diverse data, a small percentage of coverage, such as 2%, may contain many undetected species [51]. Classic sample completeness is defined as the observed richness divided by the estimated true species richness. Since true richness typically cannot be accurately estimated for species data, classical sample completeness cannot be accurately estimated from the sample itself. In contrast, sample coverage, originally developed by the founder of computer science, Alan Turing, can be accurately estimated from the sample itself. For example, if we assume that we have three dominant species in a community, their proportions of individuals in the total assemblage are 50, 30 and 15%, respectively, and there are 100 other species, each representing only 0.05%. If we take three observations and assume that three dominant species are observed, the sample coverage of this sample would be 95%, whereas the classical sample completeness would be 3/103 (approx. 3%). A very small percentage of coverage can contain many species due to the possible presence of vanishingly rare species. In this example, 5% coverage includes 100 species, whereas 95% includes only three species. Therefore, whether a coverage value is ‘relatively’ high depends on the distribution of species abundance or reads. In our case, 98% coverage is relatively low.

To calculate the estimated taxonomic and phylogenetic diversity, we used the *estimate3D* function. The *estimate3D* function allows the standardization of diversity measures to a given sample coverage, thus controlling for differences in sample coverage. We chose a level of 99.6% sample coverage, the mean sample coverage in our data, to avoid diversity values that are mainly based on extrapolation. To test the robustness of our results, we additionally calculated all diversity measures for a standardized sample coverage of 98%, the lower range within our data. The results for a sample coverage of 98% are in electronic supplementary material, table S1.2.

We calculated taxonomic and phylogenetic diversity along Hill numbers *q* = 0, *q* = 1 and *q* = 2 to consider relative read distributions. The interpretations of taxonomic diversity (Hill number) of orders *q* = 0, 1 and 2 are the following: for *q* = 0 all species are counted equally without considering their relative frequency, hence diversity measures for *q* = 0 are more sensitive to rare species. For *q* = 1, each species is weighted in proportion to its frequency, which lays the focus on common or typical species. For *q* = 2, abundant or frequent species are weighted disproportionately, hence diversity measures for *q* = 2 represent the effective number of very abundant or dominant species. Similar interpretations are valid for phylogenetic diversity of orders *q* = 0, 1 and 2 by replacing ‘species’ with ‘lineages’.

### (i) Generalized additive models

Analyses of the diversity measures are based on models from Uhler *et al*. [32]. We used the *gam* function from the *mgcv* package [52] to fit generalized additive models to test for the effects of land use and climate on taxonomic and phylogenetic diversity along the Hill numbers for each sample. Taxonomic and phylogenetic diversity values were kept as decimal numbers and modelled with a negative binomial error term (R scripts 7 and 8). Predictors for land use included local land-use types (forest, grassland, arable land and settlement) and regional land-use types (semi-natural, agricultural and urban). The mean day of a trap-specific sampling period was modelled by a smoothed non-linear spline of time to account for seasonality and an offset for sampling length to control for variation in individual sampling periods. A correlated plot-specific intercept (geographical location of the plot) was used to account for the spatial arrangement within and between grids and for repeated measurements per plot. Mean annual temperature and precipitation served as predictors of macroclimate. The average local temperature and humidity for each plot were determined based on the specific sampling period of each insect collection. Furthermore, we fitted generalized additive models to test for effects of land use and climate on sample coverage. The model included the same variables as mentioned above but a binomial error term. Significant differences between land-use categories were assessed by multiple post hoc comparisons using the *glht* function from the *multcomp* package [53]. Calculations of generalized additive models and post hoc comparisons can be found in R scripts 7 and 8 for taxonomic and phylogenetic diversity, respectively. All model results for a standardized sample coverage of 99.6% are given in electronic supplementary material, table S1.1. Additional results for a standardized sample coverage of 98% are given in electronic supplementary material, table S1.2.

## 5. Results

**Figure 3.**
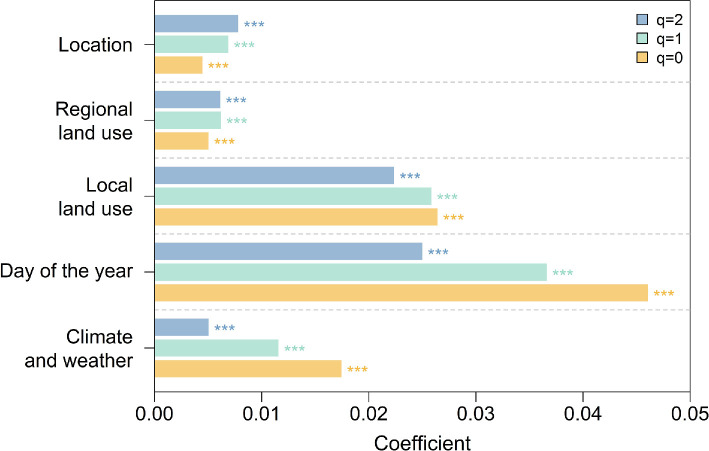
Model coefficients of multiple regressions on distance matrices, calculated with focus on rare (*q* = 0), common (*q* = 1) and dominant (*q* = 2) species. Models included geographic distances (based on geographic coordinates in m), distances of regional and local land-use categories, day of the year and climate and weather. Models were calculated for coverage standardized dissimilarity matrices along the Hill numbers (*q* = 0, *q* = 1, *q* = 2) to control for relative frequency distributions of reads generated during metabarcoding. Significant results (*p* ≤ 0.001) are marked with three stars.

The results of the multiple regressions show that the day of the year had the strongest effect on community composition (regression coefficients: 0.046 (*q*0), 0.037 (*q*1), 0.025 (*q*2)) followed by local land-use types and climate and weather. Regional land use and geographic location had the smallest impact (figure 3). In addition, climate and day of the year (i.e. mean date from the timeframe at which the insects were sampled) had stronger effects on communities with a focus on rare (*q*0) and common species (*q*1).

Sample coverage was significantly higher in arable land compared to forests and in urban regions compared to semi-natural regions (electronic supplementary material, table S1.1). Sample coverage was also affected by the day of the year, with the lowest sample coverage occurring in July (electronic supplementary material, table S1.1 and figure S1.2).

**Figure 4.**
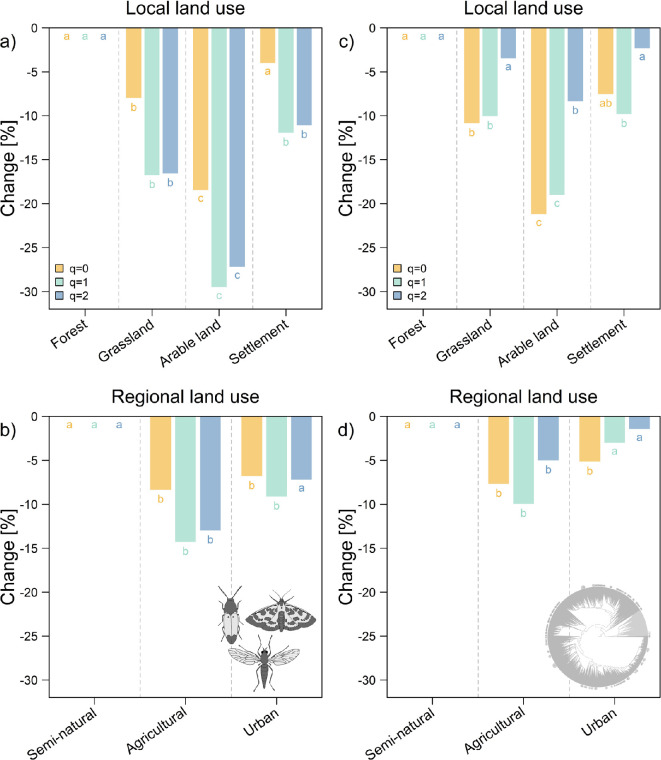
Multiplicative partial effects of land use on taxonomic and phylogenetic diversity based on generalized additive mixed models. Models included local and regional land-use categories and controlled for season, climate, weather and space. Taxonomic diversity values were calculated for a standardized sample coverage of 99.6%. The values are in comparison to (a) the local land-use type forest and (b) regional land-use type semi-natural. Models were calculated along the Hill numbers (*q* = 0, *q* = 1, *q* = 2) to control for relative read distributions. We display all model parameters in electronic supplementary material, table S1. Letters indicate significant differences between categories (*p* < 0.05).

Model results of the sample coverage-controlled diversity values showed that forests and semi-natural regions had the highest values of taxonomic (figure 4a,b) and phylogenetic (figure 4c,d) diversity. The negative effects of local and regional land use in comparison to forest and semi-natural environments were strongest for arable land and agricultural regions (for taxonomic diversity and *q*1: Est.: −0.35, *z*: −9.67, *p* < 0.001 and Est.: −0.15, *z*: −4.63, *p* < 0.001; estimated degrees of freedom for mean day of the year: 8.68 and for geographic coordinates: 5.52).

Within taxonomic diversity, common (*q*1) and dominant (*q*2) insect species showed the strongest negative responses to land use. These effects occurred in grassland, arable land and settlements (figure 4a) as well as in agricultural regions (figure 4b). In contrast, taxonomic diversity in urban regions was only significantly reduced focusing on rare (*q*0) and common species (*q*1; figure 4b).

The results for phylogenetic diversity followed the same patterns as taxonomic diversity regarding the different land-use types. Still, reductions in phylogenetic diversity were most pronounced for rare and common species (figure 4c,d).

## 6. Discussion

Using our newly developed workflow, we quantified for the first time the impact of land use on both taxonomic and phylogenetic insect diversity across a broad range of terrestrial insect families captured by Malaise traps, with standardized sample coverage.

Our workflow integrates a singleton filter to refine metabarcoding read frequency distributions, a pipeline for constructing a phylogenetic tree from COI sequences and a standardized approach for analysing metabarcoding data. This enables the consistent calculation of taxonomic and phylogenetic diversity along the Hill numbers framework using iNEXT, opening new avenues for robust biodiversity assessment. In summary, our approach allows metabarcoding data to be analysed using the latest statistical and mathematical methodologies.

We argue that metabarcoding reads can serve as a reliable approximation of abundance, provided that standardization is performed within rather than across samples and that taxa are not directly compared. A major concern in previous studies has been that sequencing errors and variations in sequencing success across taxa and samples could bias read frequencies. As a result, many ecologists have opted to remove singletons from their data. However, from a mathematical perspective, this is problematic: removing singletons not only leads to the loss of real rare taxa but also invalidates statistical methods that rely on singleton counts [18]. To address this issue, we implemented a filtering step to remove unreliable reads while ensuring that singletons remain mathematically robust. Additionally, we calculated sample coverage within each sample to account for potential biases introduced by variations in trapping efficiency and sequencing depth. Size sorting further improved sequencing success across all size classes, strengthening the correlation between biomass and read counts [34]. Since our study focuses on overall insect diversity rather than direct taxon comparisons, differences in sequencing efficiency between species do not influence our results. Taken together, these methodological refinements provide strong support for the use of read frequencies as a reliable proxy for abundance.

### (a) Sample coverage varies between habitat types

Sample coverage varied over the sampling period and was influenced by local and regional land-use types. The highest sampling coverage was observed in arable land and urban regions (electronic supplementary material, table S1.1), indicating insect communities in these environments can be simplified. This suggests that with equivalent sampling efforts, a greater proportion of the insect communities can be effectively surveyed in these heavily anthropogenically transformed ecosystems compared to ones that are more natural. Conversely, the diversity observed in natural environments, such as forests, which generally exhibit lower sampling coverage, is underestimated relative to these simplified environments. Sample coverage was also affected by the day of the year (electronic supplementary material, table S1.1 and figure S1.2), with the lowest sample coverage occurring in July. During that time, we can also observe the highest biomass values, as shown in Uhler *et al*. [32]. These results show that we face the following two sources of sampling incompleteness in insect community studies: underestimation of diversity in species-rich habitats or landscapes, and underestimation within large samples, which contain huge amounts of biomass leading to increasingly undetected singletons [17]. While further development of sequencing depth [36] could increase the coverage within large samples, the bias in sample coverage caused by different environments will remain. This affects even conventional species-abundance data from selected orders or families [12,54]. We therefore strongly advocate standardization of sample coverage, not only when comparing different habitat types but also when comparing data within or between years, to avoid systematic sampling bias in diversity measures [13,17].

### (b) Common and dominant species react strongest in agricultural environments but not in urban regions

Forests and semi-natural regions showed the highest values of taxonomic (figure 4a,b) and phylogenetic (figure 4c,d) diversity. The negative effects of local and regional land use were strongest for arable land and agricultural regions, with 18–30% and 8–14% less taxonomic insect diversity (OTUs), respectively, compared to forests. These results are in line with Uhler *et al*. [32], who showed similar trends for barcode index number richness. It also confirms the general impression that agricultural areas have an impoverished biodiversity even when focusing on species from all families of insects [2,55,56].

By considering different frequency distributions of species via the Hill numbers, we were able to show that common and dominant insect species showed the strongest negative responses to land use. These effects occurred in grassland, arable land and settlements, as well as in agricultural regions (figure 4). Similar responses of highly abundant species were observed for population trends of hoverflies [57] and also for a broader selection of terrestrial insects over time [12,58]. These newly observed trends are in contrast to previous expectations of rare species or species with small range size being especially prone to population declines [59,60].

In contrast, taxonomic diversity in urban regions was only significantly reduced for rare and common species (figure 4). Although urban environments generally have a negative impact on insect populations [58,61], e.g. through habitat loss due to soil or surface sealing, they are still more heterogeneous and complex than agricultural areas [62] and may therefore elicit more distinct responses from different taxa [63–65]. Our results complement recent studies showing that temporal declines in insects are also most pronounced in dominant species [12]. However, while the aforementioned meta-analysis [12] found that the temporal negative trends in diversity disappeared after standardization for sample coverage, the negative effects of more intensive land use in our study persisted after standardization.

### (c) Responses of phylogenetic diversity are driven by rare species

While it is widely accepted in the scientific community that phylogenetic diversity is an important aspect in nature conservation [66,67], the comprehensive inclusion of phylogenetic information in metabarcoding has been limited by the lack of available phylogenetic trees. Our new approach to calculate phylogenetic trees with sequences employs an existing backbone tree, thereby facilitating the rapid and straightforward application to large metabarcoding data, a step forward from previous approaches, such as that of Chester [28]. It also allows us, for the first time, to analyse phylogenetic responses of such a broad range of insects identified by metabarcoding and in a consistent framework directly comparable with taxonomic diversity.

The results for phylogenetic diversity were similar to those for taxonomic diversity but more pronounced for rare and common species. We observed the greatest reduction in phylogenetic diversity in arable land for *q*0 and *q*1 (approx. 20%; figure 4c). These findings suggest that species loss in agricultural areas does not occur randomly across the phylogeny. The weaker response of dominant species in terms of phylogenetic diversity indicates that larger lineages, such as orders or families, persist in these areas. In contrast, when focusing on species richness (*q*0; i.e., all OTUs regardless of their abundance or frequency), we observe stronger effects on phylogenetic diversity, implying a non-random reduction in younger sections of the phylogenetic tree, such as genera. This aligns with a study on red-listed beetles, which also found a strong phylogenetic signal for the extinction risk of species [68].

Focusing on common species (*q*1, Shannon diversity), we observed strong reductions in both taxonomic and phylogenetic diversity. These results are quite alarming. Since a reduced phylogenetic diversity can also lead to less stability in a community [26], insect declines in agricultural environments might also accelerate in the future. The differences in Hill numbers also highlight the importance of considering read or frequency distributions in biodiversity analyses, since they can affect community responses and their extent of responses quite heavily.

## 7. Conclusion

Our findings indicate for the first time that the negative effects of land use on total insect taxonomic diversity are more pronounced in common and dominant species than in rare species. Moreover, these negative effects persisted even after accounting for differences in sample coverage. In contrast, the decline in phylogenetic diversity was significant across both rare and common species, highlighting the importance of incorporating phylogenetic perspectives into conservation strategies. Furthermore, the strong negative responses in phylogenetic diversity indicate that ecosystem stability might be more affected than taxonomic responses alone would imply.

Taken together, our findings suggest that in temperate regions, such as Central Europe, land-use intensity is the primary driver of overall insect diversity decline. However, a deeper understanding of interactions with climate and weather, including their impact on the extinction risk of individual species, remains elusive. Long-term studies incorporating both climate and land-use gradients—such as those used in this study—are still scarce, and there is a critical need for replicated land-use change experiments with appropriate controls.

## Data Availability

All data, code and materials used in the analysis are available at Zenodo (data [69]; code [38]). Supplementary material is available online [70].
